# BRAZILIAN GASTRIC CANCER ASSOCIATION GUIDELINES (PART 2): UPDATE ON TREATMENT

**DOI:** 10.1590/0102-672020210001e1563

**Published:** 2021-05-14

**Authors:** Leandro Cardoso BARCHI, Marcus Fernando Kodama Pertille RAMOS, André Roncon DIAS, Nora Manoukian FORONES, Marineide Prudêncio de CARVALHO, Osvaldo Antonio Prado CASTRO, Paulo KASSAB, Wilson Luiz da COSTA-JÚNIOR, Antônio Carlos WESTON, Bruno ZILBERSTEIN, Consenso, Álvaro Antônio Bandeira Ferraz, Amir ZeideCharruf, André Brandalise, André Maciel da Silva, Barlon Alves, Carlos Augusto Martinez Marins, Carlos Alberto Malheiros, Celso Vieira Leite, Claudio José Caldas Bresciani, Daniel Szor, Donato Roberto Mucerino, Durval R. Wohnrath, Elias JirjossIlias, Euclides Dias Martins, Fabio PinatelLopasso, Felipe José Fernandez Coimbra, Fernando E. Cruz Felippe, Flávio Daniel Saavedra Tomasisch, Flavio Roberto Takeda, Geraldo Ishak, Gustavo Andreazza Laporte, Herbeth José Toledo Silva, Ivan Cecconello, Joaquim José Gama Rodrigues, José Carlos Del Grande, Laércio Gomes Lourenço, Leonardo Milhomem da Motta, Leonardo Rocha Ferraz, Luis Fernando Moreira, Luis Roberto Lopes, Marcelo Garcia Toneto, Marcelo Mester, Marco Antônio Gonçalves Rodrigues, Maurice Youssef Franciss, Nelson AdamiAndreollo, Oly Campos Corletta, Osmar Kenji Yagi, Osvaldo Malafaia, Paulo Pimentel Assumpção, Paulo Roberto Savassi-Rocha, Ramiro Colleoni, Rodrigo Jose de Oliveira, Rubens Antonio AissarSallun, Rui Weschenfelder, Saint Clair Vieira de Oliveira, Thiago Boechat de Abreu, Tiago Biachi de Castria, Ulysses Ribeiro, Williams Barra, Wilson Rodrigues de Freitas

**Affiliations:** 1 Hospital das Clinicas HCFMUSP, Faculty of Medicine, University of São Paulo, São Paulo, SP, Brazil; 2 Faculty of Medicine São Leopoldo Mandic, Campinas, SP, Brazil; 3 Department of Surgery, Santa Casa de São Paulo, São Paulo, SP, Brazil; 4 Department of Abdominal Surgery, AC Camargo Cancer Center, São Paulo, SP, Brazil; 5 Department of Medicine, Baylor College of Medicine, Houston,Texas; 6 Department of Surgery, Santa Casa de Porto Alegre, Porto Alegre, RS, Brazil

**Keywords:** Gastric cancer, Practice cuideline, Gastrectomy, Lymphadenectomy, Combined modality therapy, Consensus, Câncer gástrico, Guia de prática clínica, Gastrectomia, Linfadenectomia, Terapia combinada, Consenso

## Abstract

**Background:**

: The II Brazilian Consensus on Gastric Cancer of the Brazilian Gastric Cancer Association BGCA (Part 1) was recently published. On this occasion, countless specialists working in the treatment of this disease expressed their opinion in the face of the statements presented.

**Aim:**

: To present the BGCA Guidelines (Part 2) regarding indications for surgical treatment, operative techniques, extension of resection and multimodal treatment.

**Methods:**

To formulate these guidelines, the authors carried out an extensive and current review regarding each declaration present in the II Consensus, using the Medline/PubMed, Cochrane Library and SciELO databases initially with the following descriptors: gastric cancer, gastrectomy, lymphadenectomy, multimodal treatment. In addition, each statement was classified according to the level of evidence and degree of recommendation.

**Results:**

: Of the 43 statements present in this study, 11 (25,6%) were classified with level of evidence A, 20 (46,5%) B and 12 (27,9%) C. Regarding the degree of recommendation, 18 (41,9%) statements obtained grade of recommendation 1, 14 (32,6%) 2a, 10 (23,3%) 2b e one (2,3%) 3.

**Conclusion:**

The guidelines complement of the guidelines presented here allows surgeons and oncologists who work to combat gastric cancer to offer the best possible treatment, according to the local conditions available.

## RESUMO

**Objetivo:**

O II Consenso Brasileiro de Câncer Gástrico da Associação Brasileira de Câncer Gástrico ABCG (Parte 1) foi recentemente publicado. Nesta ocasião inúmeros especialistas que atuam no tratamento desta doença expressaram suas opiniões diante declarações apresentadas.

Apresentar as Diretrizes da ABCG (Parte 2) quanto às indicações de tratamento cirúrgico, técnicas operatórias, extensão de ressecção e terapia combinada.

**Métodos:**

Para formulação destas diretrizes os autores realizaram extensa e atual revisão referente a cada declaração presente no II Consenso, utilizando as bases Medline/PubMed, Cochrane Library e SciELO, inicialmente com os seguintes descritores: câncer gástrico, gastrectomia, linfadenectomia, terapia combinada. Ainda, cada declaração foi classificada de acordo com o nível de evidência e grau de recomendação.

**Resultados:**

Das 43 declarações presentes neste estudo, 11 (25,6%) foram classificadas com nível de evidência A, 20 (46,5%) B e 12 (27,9%) C. Quanto ao grau de recomendação, 18 (41,9%) declarações obtiveram grau de recomendação 1, 14 (32,6%) 2a, 10 (23,3%) 2b e um (2,3%) 3.

**Conclusão:**

O complemento das diretrizes aqui presentes possibilita que cirurgiões e oncologistas que atuam no combate ao câncer gástrico possam oferecer o melhor tratamento possível, de acordo com as condições locais disponíveis.

## INTRODUCTION

It is undeniable that the multimodal treatment of gastric cancer (GC) has provided great advances to patients in many aspects. For instance, increasing overall survival (OS), increasing disease-free survival (DFS), quality of life improvement, among others. The multimodal treatment is known as the association of surgical treatment with other treatment modalities such as neoadjuvant, perioperative, adjuvant chemotherapy (CHT), radiotherapy (RT) and immunotherapy. Even with the evident evolution in relation to the new treatment regimens, such as new drugs which provides better patient tolerance and less toxicity, no chemoradiotherapy (CHRT) or immunotherapy can replace a surgery performed properly. In this sense, excluding early tumors that meet the inclusion criteria for endoscopic resection, free margins gastric resection associated with extended lymphadenectomy (R0 resection) remains the only possibility of cure^2^.

The progress of surgical treatment over the past decade is also unquestionable. The adoption of laparoscopic surgery as an alternative treatment approach, the implementation of new technologies such as the robotic platform, the earlier oral intake, more aggressive resections (multivisceral resection and super-extended lymphadenectomy) and the reduction in postoperative complications are some of the examples that corroborate this improvement^55^.

Recently, the Brazilian Gastric Cancer Association (ABCG) published the II Brazilian Consensus on Gastric Cancer^5^ and the diagnosis, staging, endoscopic treatment and follow-up guidelines. In these two opportunities, new concepts were incorporated, and old knowledge was updated. This second part of the ABCG Guidelines aims to explain the statements regarding GC treatment presented in the II Consensus.

## METHODS

For the preparation of this study, the authors used the Medline/PubMed, Cochrane Library and SciELO bases. The following descriptors were used: gastric cancer, gastrectomy; lymphadenectomy; multimodal treatment. Comments on statements about GC treatment were made based on the most recent studies and with the best statistical relevance. In addition, each statement was classified according to the level of evidence and the degree of recommendation adapted from the of the Brazilian Medical Association/Federal Council of Medicine Guidelines. More details on the applied methodology can be found in the II Consensus and in the Guidelines Part 1^5^. Note that the numbering of the statements is not in sequential order. They were divided according to the topic in question.

## RESULTS

Of the 43 statements present in this study, 11 (25,6%) were classified with level of evidence A, 20 (46,5%) B and 12 (27,9%) C. Regarding the degree of recommendation, 18 (41,9%) statements obtained grade of recommendation 1, 14 (32,6%) 2a, 10 (23,3%) 2b e 1 (2,3%) 3.

### Surgery statements

#### Statement 16

For tumors that do not meet endoscopic resection indication (T1b), surgery is indicated. In these cases, the recommended lymph node dissection is the removal of the perigastric lymph nodes (D1) in well-differentiated tumors smaller than 1.5 cm and associated with the removal of some lymph nodes in the N2 chain (D1+) for undifferentiated tumors smaller than 1.5 cm. 76% Agreement (level of evidence B; degree of recommendation 1).

#### Comments

The tumors at an early stage (early GC) whom are not included in the extended indications of endoscopic resection should be treated with free margins gastrectomy and removal of regional lymph nodes. According to the Japanese guidelines, well-differentiated T1b lesions <1.5 cm, the recommended treatment is D1 gastrectomy; T1b lesions larger than 1.5 cm, well differentiated, the recommended treatment is D1+ gastrectomy. In this situation, subtotal or total gastrectomy is associated with 98% 5-year recurrence-free survival^65^.

#### Statement 17

In stage IB-III tumors (T2-4 any N), D2 lymph node dissection is indicated. 98% Agreement (level of evidence A; degree of recommendation 1).

#### Comments

The extension of lymphadenectomy has been the subject of great debate around the world in recent decades, especially in western countries. To clarify the prognostic relevance of extended lymphadenectomy, some randomized clinical trials (RCT) were performed comparing the type of lymphadenectomy (D2 vs. D1). Among the studies with good statistical relevance and sufficient sampling are the Dutch and British studies. These trials failed to demonstrate the benefit of D2 lymphadenectomy due to the high operative mortality in the D2 group (9.7% in the Dutch study and 13.5% in the British), which reflected a sharp decline in the survival curve of this group in the initial follow-up period (45% 5-year survival in D1 group vs. 47% in D2 group in the Dutch trial and 35% in D1 group vs. 33% of D2 group in the British one). In fact, it was shown that the highest mortality rate and surgical complications with D2 dissection were mainly related to distal pancreatectomy and/or splenectomy, which were then included during standard D2 lymphadenectomy and considered, at that time, necessary for adequate lymph node dissection^14^. Finally, the long-term follow-up (15 years) of the Dutch trial revealed that D2 lymphadenectomy was associated with lower rates of locoregional recurrence and GC related death when compared to D1 dissection^59^. Indeed, surgical mortality has declined considerably in the Netherlands and the United Kingdom due to surgical training and the centralization of patients in high-volume hospitals. The lead researcher in the British trial declared that the results of his study are no longer sustainable arguments against D2 gastrectomy these days^14^. In light of such evidence and with the acquired surgical experience, the modern western point of view on the extension of lymphadenectomy has changed radically, now in an international agreement: D2 lymphadenectomy is recommended as the standard treatment with curative intent for advanced GC. 

#### Statement 21

Lymphadenectomies more extended than D2 (D2+ or D3) should be reserved only in selected cases. 92% Agreement (level of evidence B; degree of recommendation 2b).

#### Comments

Gradually, the term D3 lymphadenectomy has been replaced by “para-aortic nodal dissection” or PAND. According to some studies, the involvement of GC metastases in this region varies from 8.5% to 28%.

The main indications for this type of lymphadenectomy are healthy patients, tumors of the upper third of the stomach and diffuse histological type. However, the systematic removal of N3 level nodes was somewhat abandoned after the results of the JCOG 9501 trial^47^. In the era of multimodal treatment for advanced GC, recent studies have shown interesting results of PAND after neoadjuvant CHT. A Japanese phase II trial showed that, in patients with bulky N stage with or without para-aortic nodes, submitted to neoadjuvant CHT with S-1 and cisplatin followed by D2+ PAND gastrectomy, the 3- and 5-years OS were 59% and 53%, respectively^61^. These impressive results are encouraging new studies of PAND associated with neoadjuvant CHT, which could bring back more aggressive lymph node dissections as a routine.

#### Statement 22

Subtotal gastrectomy should be performed on distal tumors or in cases where the proximal margin is at least 5 cm between the tumor and the esophagogastric transition. 96% Agreement (level of evidence C; degree of recommendation 1).

#### Comments

The extent of gastrectomy, whether total or subtotal, is closely related to the surgical margins. According to Japanese guidelines, the surgical margins to be considered are: T1 (at least 2 cm proximal margin), T2 (at least 3 cm proximal margin); T3/T4 (at least 5 cm). It is worth mentioning some special situations: in proximal lesions of the esophagogastric transition (Siewert II or III), smaller margins are admitted as long as confirmed by frozen biopsy; in multicentric lesions, whether early or advanced, it is recommended to investigate the CDH1 gene mutation. In these cases, it is necessary to perform total gastrectomy, regardless the tumor location, ensuring the resection of the entire gastric mucosa^2^ ^,^ ^10^.

#### Statement 23

In diffuse tumors, a proximal margin of at least 8 cm is recommended. 72% Agreement (level of evidence C; degree of recommendation 1).

#### Comments

The histological type definition is important since neoplasms with poorly cohesive cells usually present a defined infiltration pattern with infiltrative growth without precise limits in the adjacent tissues and this data alone has been related as an independent prognostic factor, including as a predictor of peritoneal recurrence. The macroscopic examination often has limitations regarding the negativity microscopic metastases, particularly in infiltrative pattern carcinomas. In order to preserve part of the stomach, frozen biopsy is mandatory^56^.

#### Statement 24

In tumors invading the distal esophagus, the resection margin must be confirmed by frozen biopsy. 94% Agreement (level of evidence C; degree of recommendation 2a).

#### Comments

Inevitably, the length of the proximal margin is closely related to the type of operation to be performed (esophagectomy or gastrectomy). Some previous studies have advocated that a margin between 5 to 12 cm is often necessary in esophagogastric transition tumors, based on the possibility of skip lesions, or tumors that are surrounded by healthy tissues. Still, a recent study has shown that smaller margins can be achieved safely when frozen biopsy is performed routinely^45^.

#### Statement 25

 Total gastrectomy is recommended for proximal tumors and early multicentric tumors. 100% Agreement (level of evidence B; degree of recommendation 2a).

#### Comments

The incidence of multicentric early synchronous GC ranges from 3% to 15% of cases. The main risk factors are male gender, advanced age, family history, atrophic gastritis, intestinal metaplasia and *H*. *pylori* infection. For this reason, whenever an early GC is diagnosed, a thorough evaluation of the entire stomach is essential for the diagnosis of synchronous lesions. The presence of multiple lesions often requires partial or total resection of the stomach (the latter is preferred, as long as the patient has adequate clinical conditions)^30^.

#### Statement 26

Splenectomy should be performed only in advanced tumors from the greater curvature of the proximal stomach, when there is invasion of the spleen or if there is evident lymph node involvement of the splenic hilum. 96% Agreement (level of evidence A; degree of recommendation 1).

#### Comments

Recently, it has been proved that, during total gastrectomy for proximal GC that does not invade the greater curvature of the stomach, splenectomy should be avoided as it increases operative morbidity without improving survival. Sano *et al.*(2017)^57^ conducted a multicenter RCT comparing the role of splenectomy in upper third tumors, without invasion of the greater curvature submitted to total gastrectomy. A total of 505 patients were included (254 with splenectomy and 251 without). The splenectomy was associated with higher morbidity and more blood loss, without increasing survival. 

#### Statement 27

Patients with unresectable or marginally resectable lesions may be candidates for conversion therapy, which consists of CHT followed by surgery to achieve R0 resection*.* 98% Agreement (level of evidence C; degree of recommendation 1).

#### Comments

The recent advances in perioperative CHT have enabled the conversion of technically unresectable or marginally resectable lesions into resectable cases. In this context, conversion surgery is indicated in cases when R0 resection can be achieved, even with the intention of cure. It is noteworthy that only about 30% of patients who begin preoperative conversion CHT are actually operated. Patients with locally advanced lesions, positive peritoneal cytology, less than three liver metastases, or metastatic interaortocaval lymph nodes (Yoshida type I and II) are the most likely to have treatment success^64^. On the other hand, the presence of more than one factor of incurability and peritoneal carcinomatosis (Yoshida type III and IV) are the patients with the worst prognosis. Surgery involves a higher frequency of associated multivisceral resections, which may impact perioperative morbidity and mortality. Retrospective studies have shown improved survival of patients undergoing conversion therapy compared to exclusive palliative treatment. Though, all these studies were retrospective, suggesting the possibility of selection bias in patients undergoing conversion therapy^54^.

#### Statement 28

Duodenopancreatectomy can be indicated in cases of locally advanced gastric cancer, T4N0-2M0, young patients and with low surgical risk. 90% Agreement (level of evidence C; degree of recommendation 2b).

#### Comments

Data concerning pancreaticoduodenectomy due to duodenal or pancreatic invasion in GC are conflicting and comes from small retrospective studies. Neoadjuvant or conversion CHT is an interesting strategy for these patients. Li *et al.*(2019)^42^ carried out a systematic review with 13 articles involving 69 patients. Postoperative morbidity and mortality were 59.4% and 1.4%, respectively. Positive cytology in the peritoneal washing was the only worst prognosis factor. The procedure carries high morbidity and mortality rates and should be reserved for clinically fit patients and only when a R0 resection can be achieved. 

#### Statement 29

Hepatectomy is indicated in infiltrative liver tumors (T4b) without peritoneal carcinomatosis. 98% Agreement (level of evidence B; degree of recommendation 2a).

#### Comments

En-bloc resection is indicated in T4b GC for clinically fit patients with no distant metastasis. The liver is not a poor prognostic factor (some authors report higher complication rates in pancreatic resections, but this is controversy and not described in hepatectomies)^46^. See statement 31 for more data on multivisceral resections.

#### Statement 30

Patients with single liver metastasis may be eligible for surgery after a multidisciplinary evaluation. 86% Agreement (level of evidence C; degree of recommendation 2b).

#### Comments

The surgical resection of liver metastasis is highly debatable. Few retrospective studies have shown long term benefit in selected patients and solitary lesion is a favorable prognostic factor. In 2017 Liao *et al*. ^43^ published a systematic review comparing the OS between hepatectomy and palliative therapy in patients with GC liver metastases. One hundred and ninety- six patients were included in the hepatectomy group and 481 in the palliative group. The average OS of patients in both arms was 23.7 and 7.6, respectively. Still, compared with palliative therapy, hepatectomy was associated with significantly lower mortality at one and two years^43^. It is important to remember that preoperative CHT is recommended in oligometastatic patients and there should be no other incurability factors.

#### Statement 31

The combined resection of adjacent or multivisceral organs can be performed as long as the patient is in good clinical condition and, preferably, R0 resection is achieved. 96% Agreement (level of evidence B; degree of recommendation 2a).

#### Comments

The multivisceral resection is indicated in the locally advanced GC that invades adjacent organs if R0 can be achieved. The survival is poor in a R1/2 setting or if metastatic disease exists. Dias *et al.*(2020)^16^ compared the rate of complications and survival between patients undergoing multivisceral resection and standard gastrectomy. More severe complications occurred more frequently after multivisceral resection (p=0.002). Surgical mortality was 8.6% and 4.9% for multivisceral resection and standard gastrectomy, respectively(p=0.221). Advanced age, comorbidities and multivisceral resection were independent risk factors for more serious complications. This study also demonstrated that DFS was lower in patients with multivisceral resection (51% vs. 77.8%; p<0.001)^16^.

#### Statement 32

In patients considered M1, palliative gastric resection may occasionally be performed in cases of obstruction, bleeding or perforation. 100% Agreement

(level of evidence B; degree of recommendation 1).

#### Comments

The palliative resection of symptomatic gastric tumors is indicated if technically feasible in patients who have the prospect of receiving some complementary quality-of-life palliative treatment. This concept has been well established since 2002 with the work of Hartgrink*et al.* ^22^, who found survival benefit after palliative resection of gastric tumors with only one metastatic site in patients under 70 years^22^. However, multivisceral resections in patients with no prospects of receiving any treatment should be avoided in order not to rush the final outcome. In clearly terminal cases, less invasive measures such as enteral feeding tube, endoscopic prostheses may be employed to relieve gastric obstruction and or even hemostatic RT in cases of tumor bleeding.

#### Statement 33

Palliative gastric resection in asymptomatic patients is not indicated as the first treatment approach*.* 86% Agreement (level of evidence B; degree of recommendation 1).

#### Comments

The resection of asymptomatic gastric tumors without the purpose of cure is defined as cytoreductive surgery. Its objective is to prolong survival or delay the onset of symptoms by performing tumor resection without the objective of achieving an R0 resection. This modality was addressed in aRCT (REGATTA) that demonstrated no benefit of its performance as a first approach of asymptomatic metastatic patients in relation to exclusive CHT treatment^18^. Doubts persist if cytoreductive surgery would be valid in a second moment in cases that had a good response to initial palliative CHT.

#### Statement 34

Partial omentectomy (up to 3-5 cm from the gastroepiploic arcade) can be performed on T1/T2 tumors and total omentectomy must be performed on T3/T4 tumors. 78% Agreement (level of evidence B; degree of recommendation 2b).

#### Comments

Traditionally, the total omentectomy is performed along with gastrectomy and lymphadenectomy in GC surgery. It is believed that total omentectomy is essential to ensure cancer cells elimination during advanced GC surgery. Differently, some retrospective studies have shown that omentum preserving surgery may not affect patient survival. There is no consensus regarding the oncological value of omentectomy in GC surgery among the European, American and Japanese guidelines. The European guidelines does not provide any guidance on this matter, while the American one recommends resection of the greater and smaller omentum in all cases. Alternatively, the Japanese guidelines recommends the preservation of the omentum 3 cm distal from the gastroepiploic vessels in T1/T2 tumors and total omentectomy in T3/T4 tumors. In fact, recent studies have shown that the incidence of metastatic involvement in the greater omentum varies from 2-5%, and the possibility of T1/T2 stage tumors are involved is practically null. Its pivotal role is not under discussion in cases where there is suspicion or evident invasion of the greater omentum^6^.

#### Statement 35

Bursectomy should be performed only on T4 tumors arising from the posterior gastric wall. 80% Agreement (level of evidence B; degree of recommendation 2a).

#### Comments

Historically, the bursectomy, which removes all omental bursa, including the anterior sheet of the transverse mesocolon, as well as the anterior capsule of the pancreas body, in addition to total omentectomy, have been advocated for local prophylactic control. The theoretical reasoning for performing bursectomy is to reduce the risk of peritoneal recurrence by removing the peritoneum that could contain micro-metastases. Conversely, prophylactic bursectomy is not routinely performed, because several other studies have not reported effects on OS in GC patients. Recently, the final results of the RCT with 1,204 patients comparing bursectomy with just the omentectomy (JCOG1001) were published. This study demonstrated that there is no difference in survival between the two groups^38^.Thus, the Japanese guidelines recommend bursectomy only for tumors involving the serous layer of the posterior gastric wall and suggests avoiding bursectomy in serosa negative tumors^2^.

#### Statement 36

Prophylactic total gastrectomy is indicated in confirmed familial hereditary gastric cancer with the CDh1 gene mutation. 100% Agreement (level of evidence C; degree of recommendation 2a).

#### Comments

Poorly differentiated histological gastric tumors, characteristic of this hereditary disease, have long been known to originate from the mucosa but commonly grow below it, through the submucosal layer, keeping the mucosa looking normal at endoscopic examination. This should be the probable reason why previously unidentified malignant lesions were found in prophylactic gastrectomy surgical specimens. This “sneaky” feature decreases the chance of early diagnosis and consequently the likelihood of curative surgical treatment, justifying the completion of prophylactic total gastrectomy. Meanwhile, we have observed in clinical practice a progressive increase of the germline mutation screening test, through genetic panel, involving the search of dozens of genes, including CDH1, for several families with apparent risk of another cancer. Even if they do not meet the clinical criteria for familial hereditary gastric cancer. In this special group of families that have the germline mutation for CDH1 but do not meet the clinical criteria, the evolution seems to be different from the classic families, with lower risk of cancer and onset of the disease at a higher middle age. Therefore, these families may need to be treated differently, at least postponing the indication of gastrectomy. Regarding the age range for surgery, complex mathematical models suggest that gastrectomy should be performed at 39 years for men and 30 years for women^39^.

#### Statement 37

Laparoscopic subtotal gastrectomy can be performed in distal third early GC. 98% Agreement (level of evidence A; degree of recommendation 1).

#### Comments

This issue focuses on changing surgical access in the treatment of early gastric adenocarcinoma of the distal third and not meeting endoscopic resection criteria. Large Korean and Japanese RCT were conducted to answer this question. In these studies, considerable rigor is described in the selection of surgical teams authorized to participate in them. The morbidity and mortality results were initially published, as expected, demonstrating not only non-inferiority, but even superiority of laparoscopic access results in part of the issues analyzed. Regarding five-year survival, these same studies have shown in later publications that laparoscopic gastrectomy is an oncological safe method^33^ ^,^ ^35^.

#### Statement 38

Laparoscopic subtotal gastrectomy can be performed in distal third advanced gastric cancer. 92% Agreement (level of evidence A; degree of recommendation 1).

#### Comments

The laparoscopic surgery in advanced tumors has always brought greater concern regarding the possibility of incomplete lymphadenectomy. Three trials JLSSG0901, KLASS-02 and CLASS-01 did not show any change in the number of early surgical complications. The analysis of short-term surgical outcomes such as resection margins, number of harvested lymph nodes and final pathological staging also did not differ between groups. Recently, the long-term survival outcomes of the CLASS-01 trial were published, confirming the preliminary data presented on the non-inferiority of laparoscopic surgery in relation to open surgery^66^. The long-term survival results of the Klass-02 study were presented in the International Gastric Cancer Congress in Prague (2019) and confirmed the non-inferiority of the laparoscopic method (data not yet published). The question whether eastern data can be extrapolated to Western patients and surgeons is always relevant. In this context, the Dutch study LOGICA serves as a bridge between the two extremes. Also, at this meeting, the initial results of this multicenter trial, which involved 10 Dutch centers and included 210 patients, were presented^24^. The preliminary data presented did not show any difference between the approaches, but these results have not yet been published. 

#### Statement 39

Laparoscopic total gastrectomy can be performed in upper third early gastric cancer. 90% Agreement (level of evidence C; degree of recommendation 2a).

#### Comments

To date, there is no RCT that confirms the non-inferiority of total laparoscopic gastrectomy over the conventional open technique for the treatment of early gastric adenocarcinoma of the proximal third. Although retrospective publications on this topic have demonstrated the safety of laparoscopic surgery compared to open surgery, it was not possible to reach the final conclusion, probably due to the small number of patients in these studies. To validate this approach, a prospective single-arm study was recently published to assess the safety of total and proximal gastrectomy in the treatment of proximal GC clinical stage I (JCOG1401). This study included 195 patients who underwent laparoscopic total gastrectomy. Grade 3 and 4 complications occurred in 29% of cases. There was no mortality, confirming the safety of the method^32^. Another Korean study (KLASS-03) also demonstrated that the incidence of morbidity after total laparoscopic gastrectomy in stage I GC did not differ significantly from that reported in a previous study for open gastrectomy^28^. The authors of both studies suggest that this approach will be one of the standard alternatives for the treatment of proximal early GC.

#### Statement 40

Laparoscopic total gastrectomy can be performed in upper third advanced GC. 76% Agreement (level of evidence C; degree of recommendation 2b).

#### Comments

Although total laparoscopic gastrectomy has been widely used in patients with advanced GC, its oncological validity has not yet been proven. To date, there are no RCT addressing the safety of this method for advanced GC. The Koreans launched the KLASS-06 trial in 2018. Nonetheless, there is no data available so far. Oh *et al.*(2020)^51^ published a meta-analysis with 3,943 patients. The operative time was longer in the laparoscopic approach and less lymph nodes were removed. However, laparoscopic gastrectomy was associated with less blood loss, shorter hospital stays and lower rates of complications. Although more lymph nodes were removed in open surgery, the overall 5-year survival was equivalent in both groups. This means that the discrepancy between the number of lymph nodes between the two approaches had no impact on the survival rate. It is concluded with these results that total gastrectomy for advanced GC can be an alternative to open surgery, yet these results need to be confirmed by randomized trials.

#### Statement 41

The use of the robotic platform has the same indications and results as laparoscopy. 90% Agreement (level of evidence B; degree of recommendation 1).

#### Comments

Outcomes related mainly to early surgical outcomes have shown that the robotic surgery is as safe and effective as the laparoscopic and open approach. However, most of these studies are unicentric and retrospective involving selected surgeons. Two recent meta-analysis including over 4,500 patients each showed that the robotic surgery has longer surgical time, less bleeding and earlier return of intestinal transit. There was no difference with respect to other early and late outcomes^9^ ^,^ ^11^. Further studies are ongoing, but no RCT have been published to date.

#### Statement 42

In Siewert type III adenocarcinomas, the standard surgery is total gastrectomy with distal esophagectomy. 96% Agreement (level of evidence B; degree of recommendation 1).

#### Comments

In Siewert type III tumors, upper mediastinal lymph node metastases are rare (1%), and very low in the inferior mediastinal region (less than 5%). The lymph node stations at higher risk for metastases among these tumors are stations 1, 2, 3, 7, and 11, besides the stations 5 and 6 (10%), which can only be adequately dissected through a total gastrectomy in these patients. Some Eastern authors advocate a proximal gastrectomy for early tumors, but this procedure has been rarely performed in our country due to its impact in quality of life related to acid reflux^23^ ^,^ ^45^.

#### Statement 43

In Siewert type II adenocarcinomas, the standard surgery is transthoracic esophagectomy (thoracoscopy) with proximal gastrectomy and gastric conduit. 64% Agreement (level of evidence B; degree of recommendation 3).

#### Comments

The standard treatment of Siewert II adenocarcinoma remains the main controversy on GEJ and gastric tumors. The literature shows and both an R0 resection and adequate lymph node dissection could be obtained either with an esophagectomy or with a gastrectomy. Both European and American retrospective studies have showed that neither surgical procedure was associated with a higher frequency of positive margins, and the U.S. Gastric Cancer Collaborative Group study has failed to identify any survival difference between the two surgeries^34^. Regarding lymph node disease, Eastern studies have demonstrated that lymph node metastases among these tumors are frequently found in paracardic nodes, in the lesser curvature nodes, and in the left gastric artery nodes, which can be dissected both in an esophagectomy and in a gastrectomy^23^. Finally, a recent systematic review has failed to identify any difference in R0 resections, lymph node dissections, anastomotic leakage, and mortality, when the two procedures were compared; this same study identified higher postoperative morbidity after esophagectomies though^25^. In conclusion, the decision must be individualized and made according to the growth behavior of the tumor (proximal or distal) and the probability of lymph node dissemination (chest or abdomen).

#### Statement 44

In Siewert type I adenocarcinomas, the standard surgery is transthoracic esophagectomy (thoracoscopy) with proximal gastrectomy and gastric conduit. 92% Agreement (level of evidence A; degree of recommendation 1).

#### Comments

The Siewert type I adenocarcinoma is associated with an incidence around 15% of upper mediastinal lymph node metastases; therefore, esophagectomy is the standard surgical treatment for these patients. The thoracoscopic procedure has been associated with a decrease in pulmonary complications compared to open surgery and should be the standard of care^8^. Nonetheless, when transthoracic esophagectomies were compared to the transmediastinal procedure, survival was improved among patients with one to eight positive nodes, even though that was a finding in a subgroup analysis^52^.

#### Statement 45

Transmediastinal esophagectomy should be reserved for patients with poor or borderline clinical conditions and/or inability to access the thoracic cavity. 78% Agreement (level of evidence A; degree of recommendation 2a).

#### Comments

According to comments in Statement 44, the approach of choice for Siewert type I is the transthoracic one. However, it cannot be considered malpractice to recommend a transmediastinal approach in patients with good performance status, in a setting where material for the thoracoscopic procedure is unavailable. Previous studies support this recommendation, as they have demonstrated a decrease in pulmonary complications when a transmediastinal esophagectomy was performed, compared to an open transthoracic procedure^27^ ^,^ ^52^. The dissection of the inferior and precarinal nodes should be mandatory though. Thereupon, a transmediastinal esophagectomy should be recommended for the cases mentioned above, if the alternative would be an open transthoracic procedure for Siewert I adenocarcinoma. 

#### Statement 46

It is recommended that following an esophagectomy, the esophagogastrostomy should be performed preferably in the cervical region. 90% Agreement (level of evidence B; degree of recommendation 2b).

#### Comments

The esophagogastric anastomosis could be cervical or intrathoracic. A European RCT and a recent Dutch multicenter study have showed that both techniques are safe if adequately performed^21^. However, the prevalent choice of a cervical anastomosis is due to the high risk of mediastinitis associated with leakage in an intrathoracic anastomosis. Mediastinitis is a complication that has a 10% mortality rate, which can be a lot higher at facilities where no immediate treatment with metallic stents is available.

#### Statement 47

Routine abdominal drain(s) are recommended for all gastric resections. 70% Agreement (level of evidence A; degree of recommendation 3).

#### Comments

The use of drains allows the removal of abdominal collections and fluids and the early diagnosis of complications such as bleeding and digestive fistulas. In no way do drains prevent these complications from occurring. In addition, thorough clinical examination supported by laboratory and imaging exams when needed can also make the same diagnosis. As a side effect, drains may predispose to local insertion site and intra-abdominal infections, cause pain impairing early ambulation, stimulate adhesion formation, and injure intra-abdominal organs.

Another utility of drains is the possibility of treating complications. In cases of bleeding, the drain prevents the accumulation of blood in the cavity that can lead to an intracavitary abscess. Digestive fistulas may have their contents emptied through the drain, avoiding diffuse peritonitis and allowing the adoption of clinical treatment. However, if any of these complications occur, imaging-guided drainage can also be performed postoperatively. Liu *et al.* (2011)^44^ published a meta-analysis with four RCTs without demonstrating any benefit in the routine abdominal drainage. The results of these studies must be carefully analyzed. The vast majority are publications from Japan and Korea, countries recognized for the high incidence of GC and for their mastery in the management of this disease. Before deciding whether or not to use a drain after a gastric resection, some aspects should be considered: the possibility of performing image-guided percutaneous drainage at any time, the surgeon’s experience and its complication rate, the volume of the hospital, the patient’s conditions and how difficult was the procedure. Anyway, data from the literature do not support its routine use.

#### Statement 48

The duodenal stump should preferably be closed using mechanical suture. 84% Agreement (level of evidence C; degree of recommendation 3).

#### Comments

The duodenal stump fistula occurs in about 5% of gastrectomy cases. The risk factors for its occurrence include distal tumors requiring greater duodenal dissection and the presence of comorbidities^53^. The widespread use of staplers made their use the choice for closing the duodenal stump. In spite of, its superiority over manual suture has been poorly studied. If there is no evidence of the best outcome regarding the occurrence of duodenal fistulas, the benefit of using staplers to decrease surgical time, especially in minimally invasive surgeries, is undeniable. 

#### Statement 49

There is no clear scientific evidence that reinforcement of the duodenal stump stapling line reduces the incidence of fistulas. 84% Agreement (level of evidence C; degree of recommendation 2b).

#### Comments

The low incidence occurrence, as the presence of numerous covariates that may influence the occurrence of duodenal stump fistula, makes the study of its association a challenge. An Italian multicenter study involving 8,268 did not show any association of reinforcement with the occurrence of fistula^12^. Kim *et al.*(2017)^37^ performed a phase II single-arm study employing laparoscopic suture to strengthen the duodenal stump without the occurrence of fistula in these cases or other related complications. Thus, the reinforcement suture still does not find justification for its use in all cases, its implementation does not add any unnecessary risk and can be performed according to the surgeon’s preference.

#### Statement 50

In subtotal and total gastrectomies, digestive transit should preferably be reconstructed by Roux-en-Y derivation. 96% Agreement (level of evidence B; degree of recommendation 1).

#### Comments

The Japanese protocol for GC treatment suggests that one of three reconstructions should be performed after total gastrectomy: Roux-en-Y esophageal jejunostomy, jejunal Interposition or double-tract method. In the case of subtotal gastrectomy, it is referred four reconstruction alternatives: Billroth I gastroduodenostomy, Billroth II gastrojejunostomy, Roux-en-Y gastrojejunostomy or jejunal interposition^51^. Comparing the Roux-en-Y reconstruction versus Billroth I and Billroth II + Braun after subtotal gastrectomy, a RCT concluded that Roux-en-Y was better in terms of frequency of biliary reflux. However, there was no difference in the postoperative quality of life index or nutritional status between these reconstruction methods^40^. Another RCT comparing Billroth I reconstruction and Roux-en-Y gastrojejunostomy after subtotal gastrectomy showed no superiority in the latter in terms of body weight loss or other nutritional aspects. In contrast, in Roux-en-Y group, reflux esophagitis and gastric stump gastritis were less prevalent (a known risk factor for cancer in the gastric remnant)^26^.

#### Statement 51

Gastrojejunostomy and esophagojejunostomy should preferably be performed with mechanical suture. 70% Agreement (level of evidence B; degree of recommendation 2a).

#### Comments

The staplers have gained great popularity because it makes it possible to perform anastomosis in a faster and more standardized way. The higher cost and unavailability of material of the appropriate dimensions are negative points. On the other hand, manual anastomosis allows the correction of the asymmetric edges of the sectioned segment during its realization, thus enabling the reinforcement of the areas identified as more friable. This fact is of great help when multiple frozen section analysis is performed. The sectioned edges do not require extensive mobilization, favoring a more tension-free anastomosis. Both techniques must be mastered in both open and minimally invasive surgery, but evidence of superiority over one another is scarce and the choice is based on the surgeon’s preference^63^.

#### Statement 52

After gastric resection, oral feeding should be started as soon as the patient has conditions and the intestinal transit is restored. 83% Agreement (level of evidence A; degree of recommendation 1).

#### Comments

The ERAS (Enhanced Recovery After Surgery) protocol recommends the early introduction of oral diet to fasten recovery and decrease postoperative complications^41^.The latest Japanese Gastric Cancer Association guidelines suggests the introduction of fluids in the 1^st^ postoperative day and solid diet between the 2^nd^ and 4^th^ postoperative days^2^. One of the great fears of the early diet introduction is intestinal distension, favoring the occurrence of anastomosis fistulas, mainly from the esophagojejunostomy. However, this risk has not been observed with the introduction of these protocols in different centers^7^. Thus, in cases where there was no intra-operative complication during the executions of the anastomosis, it is recommended to introduce an oral diet as soon as bowel transit are restored.

### Chemoradiotherapy statements

#### Statement 53

Perioperative chemotherapy (before and after surgery) is indicated for stage ≥IB resectable tumors of the distal third. 68% Agreement (level of evidence B; degree of recommendation 2b).

#### Comments

Perioperative CHT gained great prominence in the western world after the publication of the MAGIC study in 2006^13^. The possibility of administering a greater number of cycles with better tolerance and tumor downstaging were the main rationales for defending this strategy. In the following years, variations of that regimen were proposed until recently the phase III FLOT 4 trial, which compared the effectiveness of the perioperative CHT regimen (FLOT4: 5-fluorouracil (5-FU), calcium folinate, oxaliplatin, docetaxel) with ECF / ECX regimen - MAGIC trial (epirubicin, cisplatin, 5-FU or epirubicin, cisplatin and oxaliplatin), emerged as the study with the best results^1^. Another highlight of this study was the quality of lymphadenectomy, which was previously highly criticized in western perioperative CHT studies but presented adequate outcomes in the FLOT4 trial. It is important to highlight the inclusion of a large number of distal esophagus tumors (DE) and the esophagogastric transition (EGJ) in both studies. Unfortunately, no large RCT including only distal tumors has been conducted to date. The FLOT4 was limited to a subgroup analysis including only gastric tumors. Currently, the European Society for Medical Oncology guidelines - ESMO Guideline 2019 and the National Comprehensive Cancer Network Guideline (NCCN) preferentially recommend perioperative strategy regardless the lesion’s location^17^ ^,^ ^49^. However, both studies do not exclude up front gastrectomy as a valid alternative. This approach even persists as one of the most used in Japan and Korea.

#### Statement 54

Perioperative chemotherapy (before and after surgery) is indicated for stage ≥I resectable tumors of the middle and proximal third. 78% Agreement (level of evidence B; degree of recommendation 2a).

#### Comments

Studies show that patients that most respond to perioperative treatment are the ones with proximal gastric tumors. Based on literature data, the NCCN, recommends perioperative CHT based on fluoropyrimidine and oxaliplatin. The FLOT regimen with four drugs due to toxicity is recommended only for patients with good clinical conditions^49^. Unlike the NCCN, the ESMO recommends the FLOT regimen as the preferred method for perioperative treatment in stage >IB resectable GC and tumors from the EGJ^17^. (More information see statement 53).

#### Statement 55

Stage ≥IB patients who underwent surgery without perioperative chemotherapy (up front surgery) have an indication for adjuvant chemotherapy. 80% Agreement (level of evidence A; degree of recommendation 1).

#### Comments

The adjuvant CHT is indicated for stage II and III patients after surgery due to the improvement in DFS and OS. Studies have been carried out to define the impact of adjuvant CHT with a cohort of patients undergoing D2 gastrectomy. The ACTS-GC trial (The Adjuvant Chemotherapy Trial of S-1 for Gastric Cancer) randomized surgery with D2 lymphadenectomy + adjuvant CHT with the drug S1 (the combination of tegafur, a 5-FU prodrug, gimeracil and otetacil). The 5-year DFS was 65% for the S1 group and 53% for patients who underwent surgery alone. The OS in the group of patients undergoing CHT after gastrectomy was 71.7% and in the group of exclusive surgery 61.1%^29^. The CLASSIC trial (Capecitabine and Oxaliplatin Adjuvant Study in Stomach Cancer) presented data very similar data, confirming the benefit of adjuvant CHT in these situations^50^.

#### Statement 56

Adjuvant radiotherapy is recommended in cases with an indication for adjuvant chemotherapy and who did not have an adequate lymph node dissection during surgery. 82% Agreement (level of evidence B; degree of recommendation 2a).

#### Comments

The results of the INT0116 trial established the efficacy of post-operative chemoradiation in patients with completely resected gastric or EGJ adenocarcinoma who have not received preoperative therapy. In INT0116 trial only 9% of patients received D2 dissection. The median OS in the surgery only group was 27 months, as compared with 36 months in the CHRT group. After a median follow-up >10 years, survival remained improved in patients treated with post-operative chemoradiation^58^. It is important to note that patients undergoing radical surgery with an adequate lymphadenectomy (D2), recent studies have shown no DFS benefit with the addition of RT associated with CHT when compared to patients who received only adjuvant CHT^36^.

#### Statement 57

Patients with metastatic gastric cancer, in good clinical condition, have an indication for palliative CHT. 94% Agreement (level of evidence A; degree of recommendation 1).

#### Comments

The systemic therapy can provide palliation of symptoms, improved survival, and enhanced quality of life in patients with metastatic GC. It is indicated in 0-2 status performance patients. The first-line systemic therapy with two cytotoxic drugs are preferred for patients with advanced disease because of their lower toxicity^17^ ^,^ ^49^. A pioneering study published in 1997 comparing palliative CHT with only best support of care (BSC) showed better OS (6 vs. 2 months) and longer disease progression period (5 vs. 2 months) for the group that received palliative CHT^20^. Currently, regimens with more than one drug are considered as first- and second-line therapy in both west and the east countries^2^ ^,^ ^62^.

#### Statement 58

Patients with metastatic gastric cancer who respond well to palliative chemotherapy and have little residual disease are candidates for conversion therapy with the aim of achieving an R0 resection. 84% Agreement (level of evidence C; degree of recommendation 2b).

#### Comments

In clinical stage IV patients, the palliative CHT represents the current standard of care. However, conversion therapy has emerged as an alternative therapy for these stage IV patients recently (see Statement 27). Ramos *et al.*(2019)^54^ described the experience of the Cancer Institute of the University of São Paulo from 2009 to 2018. There were 113 patients with advanced GC who underwent surgery with curative intent after CHT + RT. In this cohort, 16 patients were considered treated with conversion surgery (1.6%). The OS in the conversion surgery group was higher when compared to the group without surgery (43.8% vs. 27%, respectively; p=0.037). The median 5-year OS for stage IV was 7 months compared to 11.3 months for the conversion surgery group. Conversion surgery is a novel concept aiming at R0 resection for originally unresectable or marginally resectable tumors after a remarkably good response to the CHT. Definitions regarding the best treatment regimen, diagnostic criteria of irresectability and which group of patients benefits from this modality are still necessary. 

#### Statement 59

Hyperthermic intraperitoneal chemotherapy (HIPEC) should be used only in research protocols, as there is still no consistent evidence of its real benefit. 86% Agreement (level of evidence B; degree of recommendation 2b).

#### Comments

Cytoreductive surgery combined with intraperitoneal hyperthermic chemotherapy (HIPEC-CSR) has a positive impact on patients with peritoneal and abdominal-pelvic neoplasms. The best results have been obtained in mucinous neoplasms of the cecal appendix and in peritoneal mesothelioma. Some studies have shown encouraging results from HIPEC-CSR in GC patients with a high risk of recurrence, as well as in patients with peritoneal metastases, including increased survival. Desiderio *et al.*(2017)^15^ published a meta-analysis with 11 RCT (10 Asian studies) and 21 non-randomized (2,520 patients) demonstrating a median survival benefit in favor of the HIPEC-CSR group (11 months) versus the control group (seven months). However, the patients who benefited the most were those who did not have peritoneal carcinomatosis. There is an ongoing phase III multicenter Western study (GASTRICHIP) to evaluate the effects of HIPEC with oxaliplatin in serosa positive GC patients and/or positive lymph nodes and/or positive cytology in peritoneal lavage, treated with perioperative systemic CHT and gastrectomy with curative intent^19^. Thus, HIPEC should not yet be recommended outside research protocols. 

#### Statement 60

Metastatic gastric cancer patients HER-2 positive are indicated for target therapy treatment (monoclonal antibody) associated with palliative chemotherapy. 92% Agreement (level of evidence A; degree of recommendation 1).

#### Comments

The treatment with trastuzumab is based on testing for Her-2 status and the combination with CHT can be considered as a standard option for patients with HER-2 positive advance GC or EGJ cancer. The ToGA trial was the first RCT that showed improved survival and established trastuzumabe with CHT as standard treatment for patients HER-2+ with metastatic gastric or EGJ cancer^4^. Another study evaluated the tolerance of oxaliplatin in combination with trastuzumab (mFOLFOX 6). The results were favorable and there was better tolerance than the cisplatin and fluorouracil regimen. This study suggests that the combination of mFOLFOX 6 with trastuzumab is an effective regimen and is well tolerated by HER-2+ metastatic GC patients^60^.

#### Statement 61

Immunotherapy for patients with metastatic gastric cancer should be used only in research protocols, as there is still no consistent evidence of its real benefit. 70% Agreement (level of evidence B; degree of recommendation 2a).

#### Comments

In September 2017, the monoclonal antibody pembrolizumab was approved as a treatment for patients with recurrent, locally advanced or metastatic gastric or EGJ cancer that express the PD-L1 protein. This approval was based on the results of the KEYNOTE-012 and KEYNOTE-059 studies (multicenter, non-randomized, open label) that selected patients who had disease progression with at least two CHT regimens^3^ ^,^ ^48^. Other studies suggest that pembrolizumab as a single agent or in combination with cisplatin and fluorouracil has anti-tumor activity and acceptable tolerance. It is indicated as the first line of treatment for PD-L1 positive patients. 

## DISCUSSION

The different aspects that encompass GC treatment remain in constant evolution. This is one of the main reasons for implementing this guideline. Excluding early tumors that can be resected endoscopically, surgical resection is the only modality that can lead to cure. Currently, there is no longer any doubt about the preponderant role of D2 lymphadenectomy as a fundamental basis for the treatment of advanced forms of GC. This concept about the predominant role of lymphadenectomy is based on lymphatic drainage studies in GC published by Jinnai et al. (1957)^31^ in the 50-60s, demonstrating that the lymphatic pathway is the main route of spread of the disease. The idea in which more extended lymphadenectomy could increase survival has been somewhat abandoned after studies showed no additional benefit. Interestingly, the main negative study (JCOG9501) excluded patients who had radiologically detected para-aortic LN metastases, which resulted in a low incidence of metastatic LN found in that region (8.5%). Perhaps it is exactly these patients who, after presenting a good response to CHT, may benefit from more extended lymph node dissections.

Another increasingly relevant aspect is minimally invasive surgery. There is already sufficient evidence to safely support its use and, why not, better results in partial stomach resections in both early and advanced tumors. As for total gastrectomy, current data indicates that it is a matter of time for the definitive evidence to be presented. It is even possible to dare that in a not-so-distant future, minimally invasive surgery (whether laparoscopic or robotic) will be the standard route in GC surgery, just as it happened in bariatric surgery.

Another evidence that seems to be increasingly consolidated is the performance of perioperative CHT for proximal tumors of the stomach and cardia. Studies have shown that patients tolerate better this type of approach when it is performed before the removal of the stomach. The question that remains is whether patients with resectable distal tumors would have the same benefit.

Finally, the II Consensus and the Brazilian Gastric Cancer Association Guidelines did not encompass promising studies such as the molecular classification of GC. This new type of classification may lead to new individualized target treatments, with better outcomes and patient’s tolerance.

## CONCLUSION

This article includes comments and guidelines regarding GC surgery and multimodal treatment (CHT and RT) reported in the II Brazilian Gastric Cancer Consensus of the ABCG. Surgeons and oncologists can rely on the information presented here to offer the best possible treatment, according to the local conditions available.
